# Multimodal Metabolomic Analysis Reveals Novel Metabolic Disturbances in Adults With Early Treated Phenylketonuria

**DOI:** 10.1002/jmd2.70010

**Published:** 2025-03-24

**Authors:** Yann Dos Santos, Patrick Emond, Ida Vanessa Doederlein Schwartz, Antoine Lefèvre, Camille Dupuy, Gabrielle Chicheri, Hélène Blasco, François Maillot

**Affiliations:** ^1^ Université de Tours, INSERM, Imaging Brain & Neuropsychiatry iBraiN U1253 Tours France; ^2^ In Vitro Nuclear Medicine Department University Hospital of Tours Tours France; ^3^ Medical Genetics Service Hospital de Clinicas de Porto Alegre Porto Alegre Brazil; ^4^ Departamento de Genetica Universidade Federal do Rio Grande do Sul Porto Alegre Brazil; ^5^ Department of Biochemistry and Molecular Biology University Hospital of Tours Tours France; ^6^ Department of Internal Medicine University Hospital of Tours Tours France

**Keywords:** adults, LC/MS, metabolomics, phenylketonuria, tryptophan metabolism

## Abstract

Phenylketonuria (PKU) is an inborn error of metabolism responsible for an accumulation of phenylalanine, which leads to cognitive and developmental disorders if left untreated. Most studies of adult PKU focus on neuropsychiatric complications, but new questions have been raised about systemic manifestations of PKU in adulthood. Fifteen adults with classic PKU with poor metabolic control and 15 matched healthy controls were recruited to compare their blood metabolomes by an untargeted multimodal approach (polar, apolar, and lipids) by LC/MS and a targeted approach to the tryptophan pathway. Targeted analysis revealed systemic serotonin hypometabolism and aberrant kynurenine metabolism, as well as potential implication of microbiota by differences in some indole compounds compared to controls. Untargeted analysis confirms previous findings regarding the TCA cycle, alanine aspartate glutamate metabolism, arginine and proline metabolism, and revealed some new metabolic perturbations such as arginine biosynthesis or glyoxylate and dicarboxylate metabolism. Future studies involving larger numbers of patients with varying degrees of metabolic control are needed to confirm these findings.

1

**Summary**

- Early‐treated classical PKU patients with poor metabolic control studied via a multimodal metabolomics approach show numerous metabolic disturbances potentially related to Glu depletion, as well as disturbances in energy and tryptophan metabolism including serotonin and kynurenine pathways.
- Microbota is potentially involved in these disturbances.

## 2 Introduction

Phenylketonuria (PKU) is one of the most common inherited metabolic diseases, with an incidence of approximately 1:24 000 in Europe [1]. It is mainly characterized by the presence of mutations in the phenylalanine hydroxylase (PAH) gene [2], an enzyme that converts phenylalanine (Phe) into tyrosine (Tyr). Dysfunction of this enzyme leads to an accumulation of Phe, resulting in cognitive and developmental impairments if left untreated. Phe accumulation can be controlled by a low‐Phe diet following newborn screening, which leads to normal development and cognitive abilities within the general average in terms of intelligence quotient. However, such a diet, which is recommended for life [3, 4], is poorly followed in adulthood. The impact of stopping the diet in adulthood is still largely unknown [5]. Today, most research focuses on the neuropsychological and behavioral complications of adult PKU patients [6]. However, a number of epidemiological studies based on databases from different countries report other peripheral disorders that merit consideration [7, 8, 9]. The pathophysiology of these disorders, which appear to be multisystemic, can be studied using metabolomics in an attempt to gain an overview of the potential disturbances that may be involved.

The use of metabolomics to study PKU is relevant in the search for new biomarkers that can be used for patients' monitoring [10, 11], particularly, nutritional [12, 13] or to improve newborn screening and/or diagnosis by multiple biomarker detection to avoid false positives [14, 15, 16, 17, 18, 19, 20]. A few studies have used metabolomics in an attempt to gain a better understanding of the pathophysiology of PKU, including a few animal studies exploring the brain metabolome [21, 22, 23], liver, and blood metabolome [21, 22]. Metabolomics has also been used as a targeted approach to study tryptophan (Trp) metabolism in PKU [24]. Cannet et al. [25] studied the lipoprotein profile and relative concentration of 24 metabolites in the plasma of adult PKU patients using a nuclear magnetic resonance (NMR) approach. Cannet et al. [26] also studied the urinary metabolome using the same approach in a cohort ranging from infants to young adults (range: 0.25—33 years). Moritz et al. [27] also studied the metabolomic profile of a cohort with a wide age range (1.6–48.6 years) using an LC–MS/MS approach. The inclusion of children and adults can be problematic as the metabolism of children and adults presents multiple differences as illustrated by this study comparing the metabolome of children and their parents [28].

One of our previous studies [29] consisted of describing the metabolome of PKU adults in comparison to controls using a multi‐platform approach including ion‐exchange chromatographic analysis of plasma and urinary amino acids (AAs), GC–MS analysis of polar metabolites from urine samples supplemented by NMR analysis for the same matrix. This approach enabled us to identify a set of metabolites indicating systemic disturbances, mainly in the metabolism of alanine (Ala), aspartate (Asp), glutamate (Glu), arginine (Arg) and proline (Pro). More recently, Weerd et al. [30] studied the metabolome and lipidome of adult PKU patients treated early with low levels of Phe and/or sapropterin, revealing numerous lipidome and metabolome perturbations in these patients including Arg and Pro metabolism. van Wegberg et al. [31] performed a targeted analysis of various Phe metabolites to correlate with cognitive function in a well‐characterized cohort of adult PKU patients; several Phe metabolites correlate with different cognitive functions, with *N*‐lactoyl‐Phe showing the best performance.

In the present study, we considered a group of adults with classic PKU in order to avoid age‐related artifacts and those related to the inclusion of other forms of the disease. The plasma samples analyzed were taken as part of a previous study on low‐grade inflammation in adult PKU [32]. In order to gain a better understanding of the pathophysiology of adult PKU, we used an untargeted metabolomics approach focusing on polar and apolar metabolites as well as lipids by LC–MS/MS in order to broaden the metabolic coverage, allowing the discovery of potential new metabolic perturbations. In addition, to further investigate inflammation in adult PKU, we performed a targeted quantitative metabolomic analysis of the Trp pathway, focusing on the kynurenine pathway and microbiota‐derived Trp metabolites (indoles). Serotonin pathway metabolites were also quantified.

## 3 Materials and Methods

### 3.1 Patients

Patients of the present study have been previously included in the INGRAPH study reported by Giret et al. [32]. Patients with classic PKU were included at the adult outpatient clinic in our reference center for inherited metabolic diseases. The inclusion criteria were: age ≥ 18 years, PKU treated early following a diagnosis confirmed by neonatal screening, and finally that the patient was affiliated to the national health insurance system. Healthy controls were included at the clinical investigation center of our university hospital. The inclusion criteria for healthy controls were: age ≥ 18 years, affiliation to the national health insurance system, and the absence of metabolic disease. The exclusion criteria for the patients and healthy controls studied were: chronic or acute inflammatory disease, current anti‐inflammatory treatment, fever at the time of inclusion, pregnancy and breast‐feeding, diabetes and hypertension, or surgery in the previous month, and legal protection measure.

### 3.2 Study Design

The experimental design is the same as the INGRAPH study [32]. In this single‐center, cross‐sectional pathophysiological study, healthy controls were matched to PKU patients for sex, BMI, and age. The study had been examined and approved by the CPP (Comité de protection des personnes) of Paris Sud Est VI and is referenced as INGRAPH on ClinicalTrials.gov (NCT04879277). All participants signed a written informed consent.

### 3.3 Bio‐Clinical and Laboratory Investigations

At inclusion, all participants in this study were weighed and measured, enabling the BMI to be calculated [32]. Age and sex, heart rate and blood pressure were also collected. Following a full clinical examination, a medical history was taken of hypercholesterolemia, certain comorbidities (alcohol/smoking) and the presence of cardiovascular disease. In the case of PKU patients, information was collected on whether they were using AA supplements (AA‐MF or glycomacropeptide [GMP‐MF]), and whether they were on a low‐Phe diet. No precise data regarding the degree of protein restriction were collected in these patients. Specifically, when referring to patients being “on diet,” this indicates the use of AA supplementation rather than adherence to a strict low‐Phe diet. None of the patients are taking sapropterin or specific Tyr supplementation. Of the patients, seven were using AA supplementation (two GMP‐MF, five AA‐MF), while eight were not receiving any supplementation. Among the patients not taking AA supplements, most had a relaxed dietary regimen before the age of 6 years (*n* = 4) or between the ages of 6 and 12 years (*n* = 3), with one patient aged between 13 and 17 years. For all samples, blood was collected at the fasting state in 1 × 6 mL EDTA tube and 1 × lithium heparin tube. To recover blood plasma, the samples were centrifuged and aliquoted before being stored at −80°C.

### 3.4 LC–MS/MS

Nontargeted analysis was performed by LC–MS/MS as described in Dupuy et al. [33]. Briefly, all samples were measured in one batch. Quality control (QC) was performed by mixing an equivalent volume of all samples. QCs were analyzed at the start of the run, every 10 samples, and at the end. To calculate signal values, Xcalibur 2.2 software (Thermo Fisher Scientific, San Jose, CA, USA) and a library of 495 standard compounds (MSML, IROA technologies) analyzed under the same conditions and with the same mobile phase gradient were used. Metabolites with a coefficient of variation greater than 30% in QC were excluded from the metabolomics analysis. Quantification of Trp metabolites was described in Alarcan et al. [34].

### 3.5 Statistical Analysis

#### 3.5.1 Multivariate Analysis

All metabolites including Trp and lipids were implemented in SIMCA 17.0.2 (Umetrics, Umeå Sweden) to analyze the merged data. The raw metabolite data were first scaled using the unit variance scaling method, also known as autoscaling (van der Berg et al. 2006). This method focuses on comparing metabolites based on correlation and has the advantage of assigning equal importance to each metabolite (van der Berg et al. 2006). A PCA Class (PCA for each group individually) was first performed to detect potential outliers (no outliers identified). Discriminant analysis (OPLSDA) was used to compare control and patient. Partial least squares (PLS) regression models were used to explain the abnormal levels of Phe and Tyr [29]. Model quality is based on several parameters: the R2X, R2Y, and Q2 values, and the CV ANOVA. The values of R2X and R2Y indicate the proportion of the variance of the x and y variables explained by the model and represent the fit of the model, while Q2 reflects the model's predictive performance [35]. CV ANOVA is used to estimate the robustness of the model. It is based on the analysis of variance tests of cross‐validated predictive residuals [29]. Variable selection is recognized for improving the predictive performance (Q2) of multivariate models [36] and enhancing the interpretation of the modeled phenomenon [37]. A common method of variable selection is Backward Q2, based on improving the Q2 prediction parameter (validated by cross‐validation, CV ANOVA) so that it is as close to 1 as possible [38]. The model with the minimum number of metabolites for a maximum Q2 was selected [38]. To improve model predictivity, we had iteratively eliminated VIPs (variable influence on projection). To eliminate them, we selected variables with low regression coefficients and wide intervals (confidence intervals calculated by the jackknife method) [38]. These were eliminated if they also had a VIP score below 1, to retain the most influential variables [39, p. 89]. This approach was applied successively until Q2 could no longer be improved. Once this stage had been completed, the validity of the OPLSDA was checked by PCA (Figure S1b) [40] and a permutation test (100 permutations) was carried out for all models to avoid over‐fitting. Once validated, the model's VIPs were used for univariate analysis. Venn diagrams were drawn (https://bioinformatics.psb.ugent.be/webtools/Venn/) to highlight the VIPs common to the different models.

Although the exclusion of Phe and its metabolites due to their aberrant concentrations is conceptually erroneous due to data preprocessing that gives equivalent weight to each variable [41], models without Phe, Phe metabolites, and Tyr are presented as Supporting Information (Tables S1–S3b) using the methodology of our previous article [29].

### 3.6 Univariate Analysis

The univariate analysis was conducted on VIPs only. The Mann–Whitney multiple test was used to compare the relative concentration of the different metabolites between patients and controls. A 1% false discovery rate according to the two‐stage step‐up method of Benjamini, Krieger, and Yekutieli was used for multiple comparisons. The Spearman test was used to assess the correlation of VIPs with Phe and Tyr according to the respective models. Bonferroni Correction was used for multiple correlation. The p‐value threshold following this correction was 0.0011 for Phe and 0.005 for Tyr. Comparisons of Trp metabolite concentrations and anthropometric data between controls and patients were made using the Mann–Whitney test. Statistical analyses were performed using GraphPad Prism 9.0.0 (Boston, Massachusetts USA).

### 3.7 Metabolic Pathways Analysis

Metabolic pathway perturbation analysis was performed using the MetaboAnalyst 6.0 web application [42]. An analysis of the VIPs of each model was carried out. The data was transformed into Log 10 and “auto‐scaled” (centered on the mean and divided by the standard deviation of each variable). This minimized the impact of noise or large variances between variables. No normalization of the data was carried out. Only pathways with an impact greater than 0.1 and Holm p less than 10^−4^ were considered, in order to focus on the pathways with the greatest impact. A Venn diagram was made to highlight the common metabolites and pathways impacted in the different models.

## 4 Results

### 4.1 Patients

Fifteen adults (mean age ± SD = 37.4 ± 8.4) with classic PKU were age and sex matched to 15 healthy subjects (mean age ± SD = 37.7 ± 7.9). There were no significant differences between the two groups in terms of age, BMI, or sex ratio (NS). Plasma Phe of the patients was 1401 ± 434.9 μmol/L (mean ± SD).

### 4.2 Metabolites

Two hundred and forty‐one metabolites were measured using the nontargeted approach. The Trp‐targeted analysis identified 20 metabolites. The two datasets were merged. After removing duplicates (Trp metabolites measured using the nontargeted approach) the total number of metabolites was 248 (Table S4).

### 4.3 OPLSDA to Compare Patients and Controls

An automatic fit was used to construct this model. SIMCA's automatic fitting extracts as many components as it deems significant. The method used to calculate the number of significant components by the software is described by Eriksson et al. [39]. The best model is based on 49 metabolites (Table S5). The performance of the model is as follows: R2X(cum) = 0.591, R2Y(cum) = 0.98, Q2(cum) = 0.961, CV ANOVA = 2.8e−17. The model was validated using a permutation test (*p* < 0.01) and PCA [40]. The score plot is shown in Figure S1.

Univariate analysis revealed a significant decrease in 39 of the 49 metabolites used in the model, and a significant increase in indole‐3‐lactic acid (I3LA) and 5 metabolites of Phe (Table S5).

### 4.4 PLS Model to Explain Phe Concentrations

**FIGURE 1:**
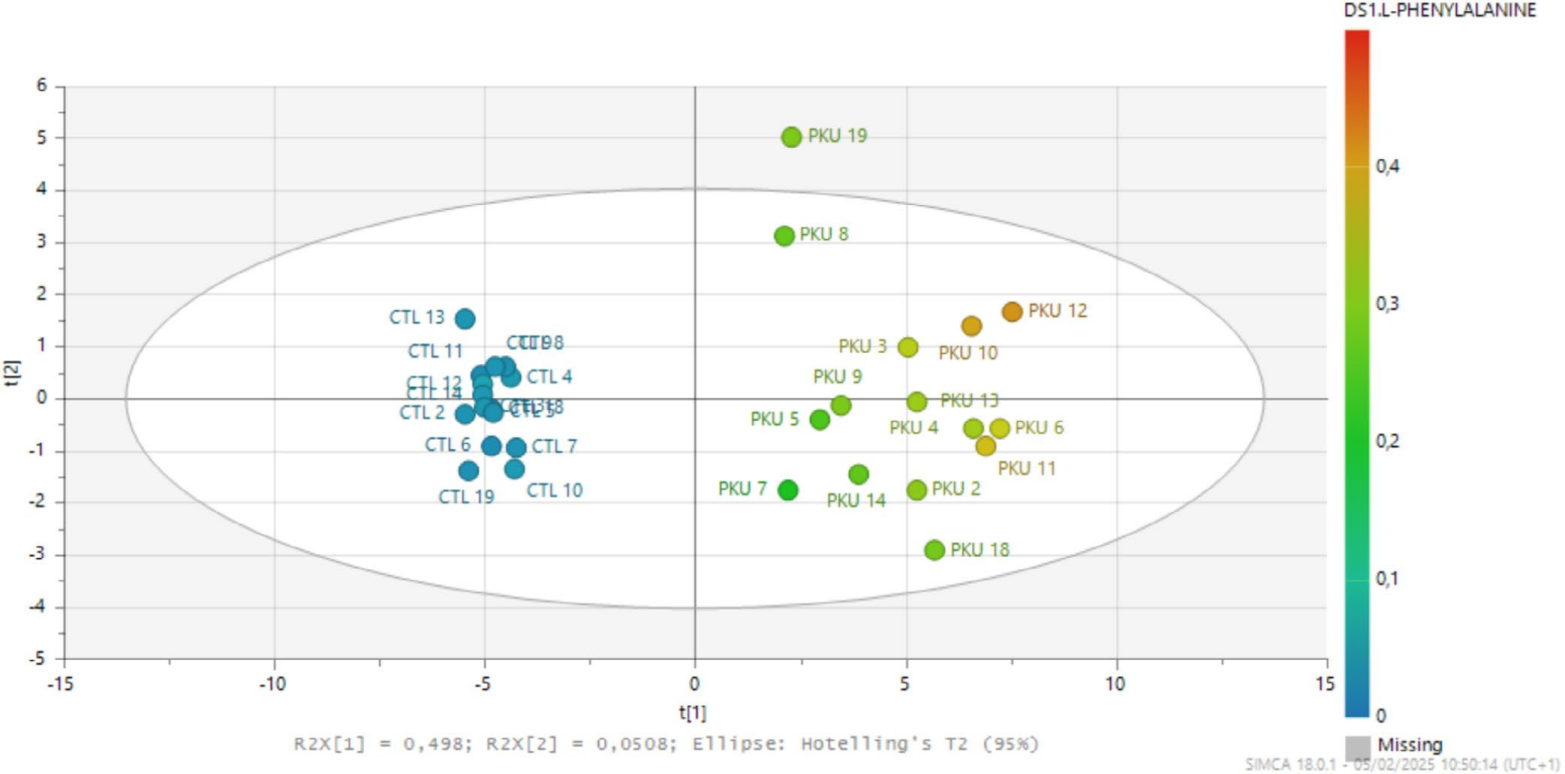
Score plot based on the best PLS model (49 metabolites) to explain Phe concentrations. The color scale indicates Phe levels. The characteristics of the model are as follows: R2X(cum) = 0.549, R2Y(cum) = 0.991, Q2(cum) = 0.978, CV ANOVA = 5.04e−20.

Score plot of the model is shown in Figure 1. An automatic fit was used to construct this model. The best model for explaining Phe concentrations was obtained from 53 metabolites (Table S6). The performance of the model was as follows: R2X(cum) = 0.549, R2Y(cum) = 0.991, Q2(cum) = 0.978, CV ANOVA = 5.04e−20.

Univariate analysis revealed a significant negative correlation between Phe and 35 of the 54 metabolites used in the model, and a significant positive correlation with I3LA, 5‐oxoproline, and 4 Phe metabolites (Table S6).

### 4.5 PLS Model to Explain Tyr Concentrations

**FIGURE 2:**
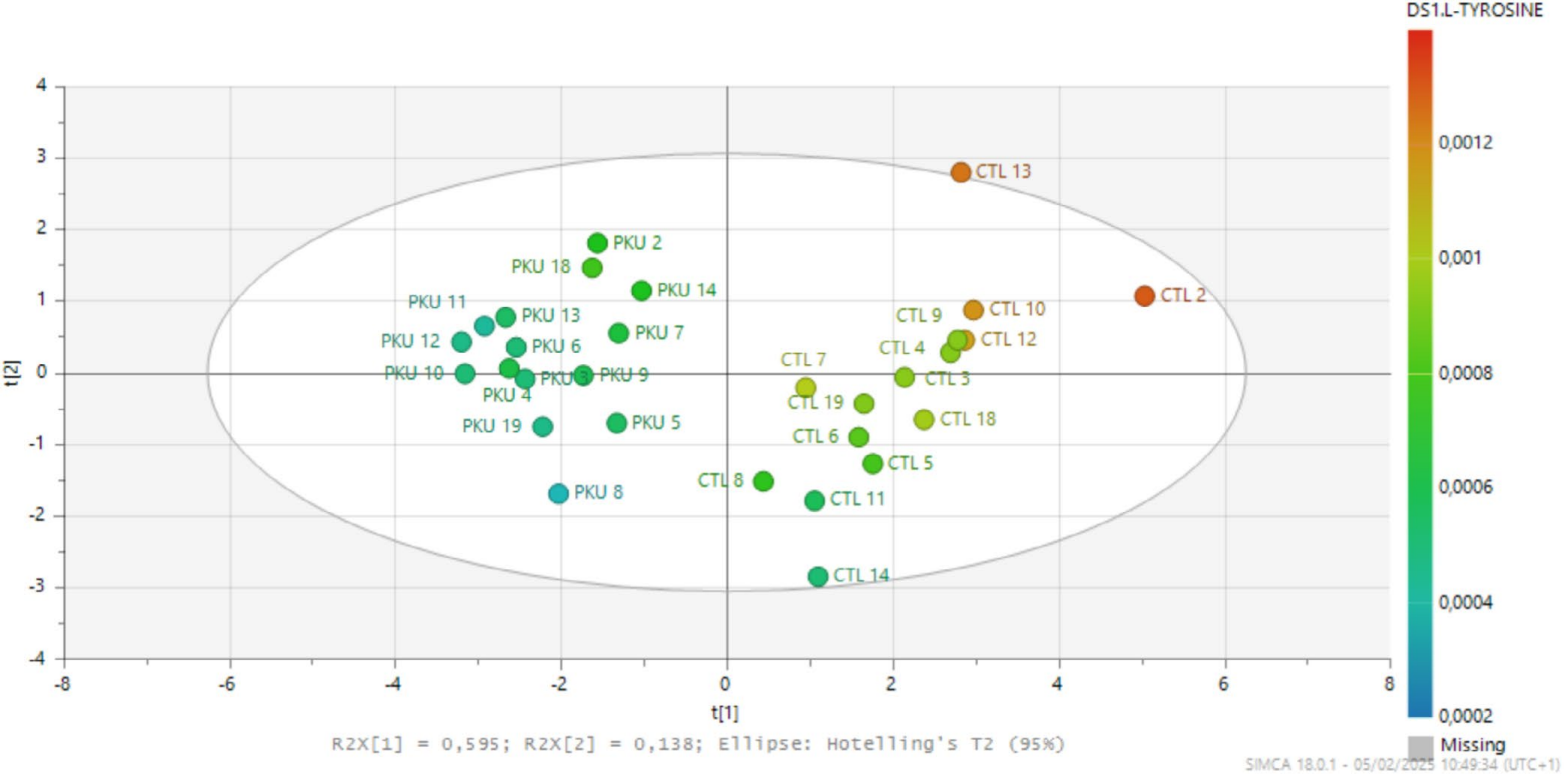
Score plot based on the best PLS model (10 metabolites) to explain Tyr concentrations. The color scale indicates Tyr levels. The characteristics of the model are as follows: R2X(cum) = 0.733, R2Y(cum) = 0.92, Q2(cum) = 0.876, CV ANOVA = 4.38709e−11.

Score plot of the model is shown in Figure 2. An automatic fit was used to construct this model. The best model to explain Tyr concentrations was obtained from 10 metabolites (Table S7). The performance of the model was as follows: R2X(cum) = 0.733, R2Y(cum) = 0.92, Q2(cum) = 0.876, CV ANOVA = 4.38709e−11.

Univariate analysis revealed a significant positive correlation of Tyr with 8 of the 10 metabolites selected, and a negative correlation with 2 Phe metabolites (Table S7).

### 4.6 Metabolic Pathways and Common Metabolites

**FIGURE 3:**
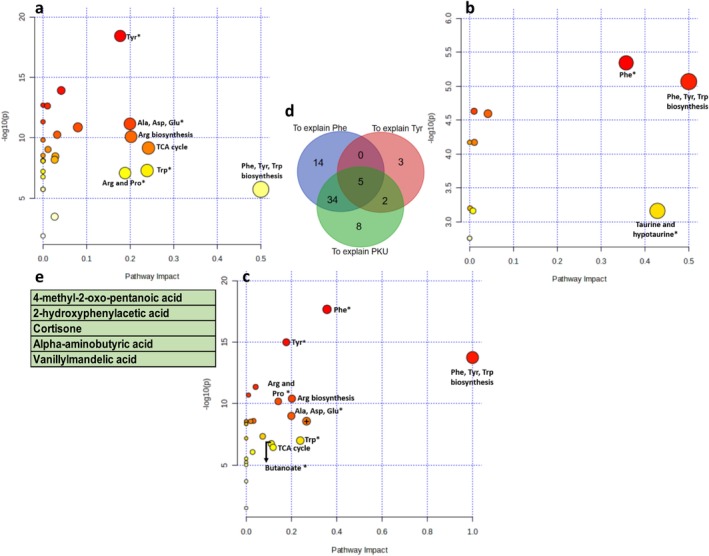
(a) Pathway analysis on PLS VIPs to explain Phe. (b) pathway analysis on PLS VIPs to explain Tyr. (c) pathway analysis on OPLS‐DA VIPs. The *y* axis represents the p‐value, the *x* axis the impact value of the channel. Node size is representative of pathway impact, node color (from white to red) is based on its *p*‐value. *, metabolism, (+) on the graph to explain PKU denotes glyoxylate and dicarboxylate metabolism. (d) Based Venn diagram showing the number of common metabolites between each model: PLS Phe on the left, PLS Tyr on the right, OPLSDA at the bottom. (e) List of five metabolites common to all three models.

The metabolic pathway analysis is shown in Figure 3a–c. Phe metabolism and Phe, Tyr, Trp biosynthesis were identified for all the models. OPLSDA and PLS to explain Phe have several other pathways in common, namely Ala, Asp, Glu metabolism, Arg and Pro metabolism, Arg biosynthesis, TCA cycle, and Trp metabolism. Pathways uniquely identified via OPLSDA are butanoate metabolism and glyoxylate and dicarboxylate metabolism (marked by + on the graph), and via the PLS to explain Tyr is taurine and hypotaurine metabolism.

The Venn diagram shows a set of five common metabolites, which are described in Figure 3e. All the metabolites shown in the diagram (Figure 3d) are presented in Figure S2.

## 5 Discussion

In our previous study [29], we used a multi‐platform approach to explore the urinary metabolome (NMR, GC–MS) and plasma AA profile (Aminotac 500 analyzer) to characterize the metabolome of adults with various forms of PKU. The aim of the present study was to explore the plasma metabolome by LC/MS using several analytical modalities (polar, apolar, lipid). First, we confirm the metabolomic signature we have identified in our previous study. However, we also detected around twofold more metabolites and discovered potential novel metabolic perturbations related to the pathophysiology of adult PKU. Indeed, our study shows some metabolic disturbances in the Trp metabolism, including both serotonin and kynurenines pathways, as well as the metabolization of Trp from microbiota. Our study also presents other findings, including numerous down‐regulated metabolic pathways in PKU potentially impacted by reduced Glu levels (fold change = 0.25) and impaired energy metabolism.

### 5.1 Metabolic Signature of Classic PKU Adults

The PLS model allowed classification of patients according to their Phe levels (Figure 1). The exclusive inclusion of patients with classic PKU avoided artifacts in patient classification associated with the inclusion of subjects with persistent moderate hyperphenylalaninemia [29]. However, we can observe heterogeneity within PKU patients. In all models, we can observe the formation of two groups corresponding to on‐ and off‐diet patients, except for patient 12 (Figure S3a–c). This patient had the highest phenylalaninemia (1784 μmol/L) of the on‐diet group, which could explain this phenomenon. Patients 8 and 19 are highly segregated (Figures 1, 2, S1b, and S2b) compared to other patients and have a Phe between 800 and 900 μmol/L and take the same AA formula. Despite poor metabolic control, diet appears to be responsible for the heterogeneity observed in these models. Patients appear to be taking their supplementation, as shown by their segregation in the different models (Figure S3a–c) representing a potentially distinct metabolic signature. We cannot assert that this is the case in the present study, as none of the metabolites differed significantly between on‐ and off‐diet patients. Studies involving larger numbers of patients with different metabolic controls and nutritional histories are needed to investigate this further.

Almost all of the discriminant metabolites for the PLS model to explain Phe were negatively correlated with Phe (Table S6), suggesting an overall influence of Phe levels on metabolism. These are mainly numerous AAs and branched‐chain AA metabolites (BCAA) pathway, metabolites of Phe, Tyr, Trp, Arg, purines, the TCA cycle, and the transport and oxidation of acyls and fatty acids (Table S6). All the pathways identified with this model are downregulated in PKU, with at least three hits per pathway. Low Tyr and downstream metabolites such as low adrenaline and low vanillylmandelic acid (degradation product of norepinephrine) indicate the possibility of endocrine and neurotransmitter disturbances in these patients. Such deficiencies have already been reported in PKU with poor metabolic control [43] but not in well‐controlled patients [44]. Endocrine dysfunction may be enhanced by low levels of cortisol (a common metabolite, fold change = 0.33), which is probably linked to low consumption of natural proteins, resulting in a potentially low intake of cholesterol [45], which is essential for corticosteroid synthesis [46]. Indeed, despite the poor metabolic control of the patients studied, they had low levels in almost all the AAs identified, which could indicate a low protein intake. This may be explained by the fact that patients who stop taking supplements or take them discontinuously may still be following a low‐protein diet [47] with potential frequent deviations explaining high Phe and decreased concentration of many AAs. Such hypotheses are reinforced by data from a plasma metabolomic study in Pah^enu2^ mice fed with a normal protein diet, which showed no plasma AA deficiency [21, 22]. Arg biosynthesis and the metabolism of Arg and Pro appear to be altered in PKU. Arg is essential for the synthesis of nitric oxide, which plays a crucial role in neurotransmission, cardiovascular function, and immune response [48]. It is of note that nitric oxide deficiency has been reported in PKU [49, 50]. Arg also regulates carbohydrate and lipid metabolism [51]. Pro metabolism influences energy status and redox balance between the cytosol and mitochondria [52]. The TCA cycle is strongly affected, as reported in PKU [53], indicating dysregulation of energy metabolism and potential oxidative stress. Ala, Asp, and Glu metabolism perturbation could be associated with TCA cycle perturbations as these AAs are important in anaplerotic reactions to ensure ATP production by mitochondria in case of lack of tricarboxylic acid [54]. Finally, we can suggest that all these changes are implicated in the clinical progression of PKU, but further studies are needed to confirm such hypotheses.

Only I3LA, 5‐oxoproline and Phe metabolites correlated positively with Phe. Similarly, the PLS model to explain Tyr allowed classification of patients according to their Tyr levels. Tyr showed a strong positive correlation with all metabolites in the model, except for Phe and 2‐hydroxyphenylacetic acid, which were strongly negatively correlated. Interestingly, the metabolism of taurine and hypotaurine was only highlighted in this model due to the low levels of taurine (fold change = 0.54). In contrast, Moritz et al. [27] reported higher levels of taurine in PKU patients. The study by Moritz et al. [27] includes both children and adults, and some of the patients studied have good metabolic control, which may explain this discrepancy. This metabolism is involved in several biological processes such as bile metabolism, antioxidant metabolism, and osmosis [55]. Taurine deficiencies can lead to multiple complications in the nervous and cardiac systems, among others [55]. Further studies of this metabolism are needed to assess its potential contribution to the pathophysiology of PKU. OPLSDA showed clear discrimination between PKU patients and controls, representing a distinct metabolomic signature in adults with PKU.

PLS and OPLSDA models had 5 metabolites in common, highlighting their potential role in the pathophysiology of PKU (Figure 3d,e). These include cortisone (steroid metabolism), vanillylmandelic acid (Tyr metabolism), 4‐methyl‐2‐oxo‐pentanoic acid (Val, Leu, Ile metabolism), alpha‐aminobutyric acid (Cys and Met metabolism, Gly, Ser, Thr metabolism), and 2‐hydroxyphenylacetic acid (Phe metabolites). The different models share no common metabolic pathways apart from Phe metabolism and the biosynthesis of Phe, Tyr, and Trp (Figure 3a–c). Some of the metabolic pathways identified had already been found in our last study, such as Ala, Asp, Glu metabolism, Arg and Pro metabolism, the TCA cycle, and butanoate metabolism [29]. Some of these pathways were reported as deficient by Lu et al. [23], such as Ala, Asp, Glu metabolism, and Arg and Pro metabolism in the cerebral cortex of Pah^enu2^ mice. Taken together, these results seem to confirm the involvement of these pathways in the pathophysiology of PKU.

### 5.2 Trp Metabolism

The Trp pathway was affected in OPLSDA and PLS to explain Phe. In the univariate analysis, it is important to note the significantly reduced level of Trp in PKU patients. However, patients remain within the reference ranges. Trp is mainly metabolized via two pathways [56]. One of these pathways is serotonin synthesis, this neuromodulator being known to be deficient in PKU [57, 58]. Despite a strong trend (*p* = 0.06), there was no significant difference for this metabolite between PKU patients and controls. In contrast, the concentrations of its precursor 5‐hydroxy‐L‐tryptophan and its degradation product 5‐hydroxy‐indole‐acetic acid were significantly reduced. Taken together, these results suggest hypometabolism. These results, although derived from plasma samples, seem to follow the trend of studies concerning the brain compartment [59]. The mechanism involved could be the low bioavailability of Trp, rapid catabolism of serotonin, a shift toward the kynurenine pathway, or competition from Phe for the catalytic site of tryptophan hydroxylases [60, 61].

**FIGURE 4:**
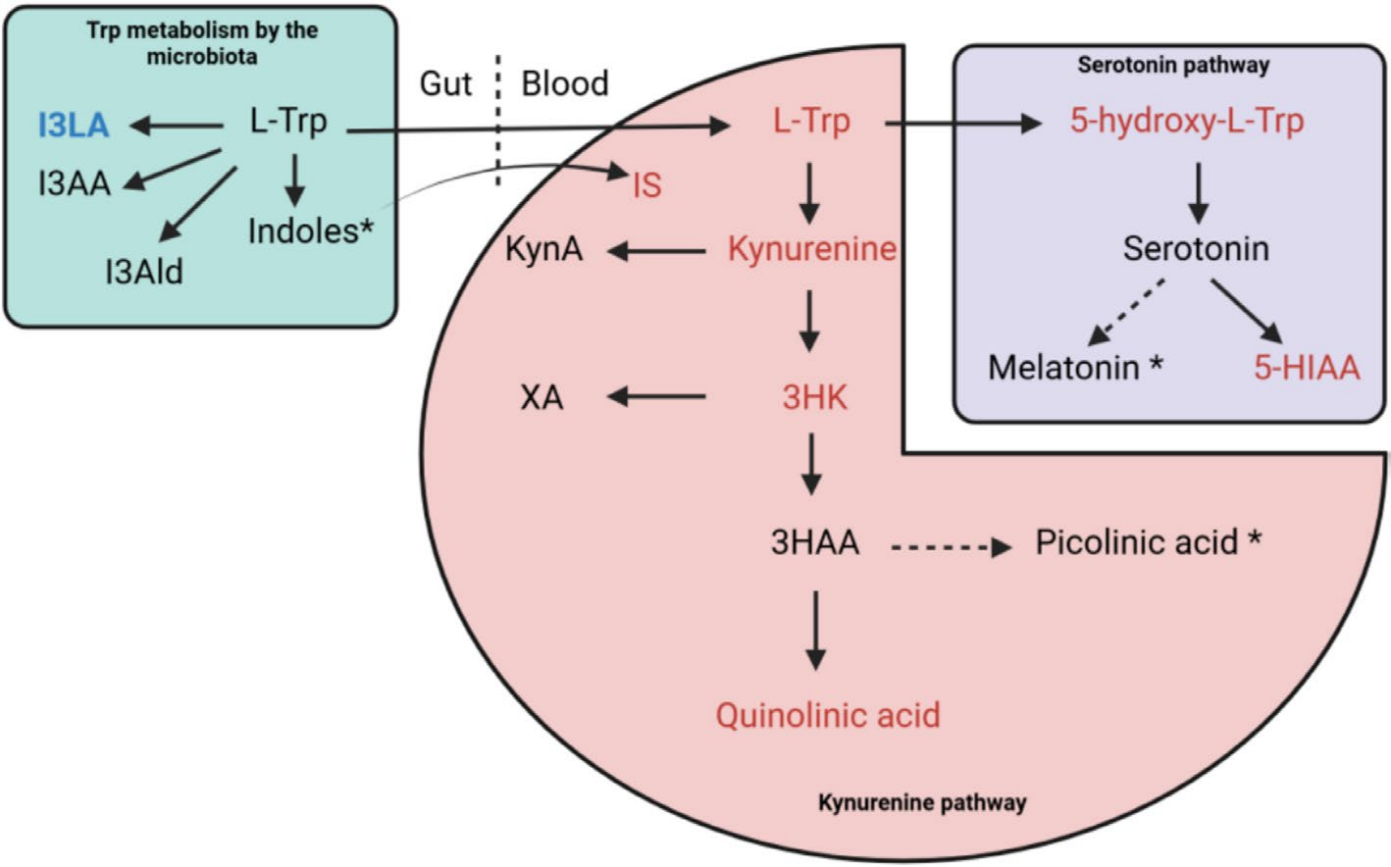
Disturbances in Trp metabolism. The red zone indicates kynurenine metabolism. The purple zone corresponds to serotonin metabolism. The blue zone refers to Trp metabolism by the microbiota. Metabolites written in black indicate no difference between control and PKU, in red metabolites significantly less present in patients, and in blue metabolites significantly more present in patients. Metabolites marked with an asterisk could not be measured in the present study. 3HAA = 3‐hydroxyanthranilic acid; 3HK = 3‐hydroxykynurenine; 5‐HIAA = 5‐hydroxyindoleacetic acid; 5‐hydroxy‐L‐Trp = 5‐hydroxytryptophan; I3AA = indole‐3‐acetic‐acid; I3Ald = indole‐3‐aldehyde acid; I3LA = indole‐3‐lactic acid; IS = indoxyl sulfate; KynA = kynurenic acid; L‐Trp = L‐tryptophan; XA = xanthurenic acid.

The other main pathway by which around 90% of Trp is metabolized is the kynurenine pathway. Its end products include quinolic acid, which ultimately regenerates NAD+ [62]. This entire metabolism appears to be deregulated in PKU. Indeed, metabolites from the main steps of the pathway were significantly less present compared to controls (kynurenine, 3‐hydroxy‐kynurenine and quinolinic acid). The intermediate pathways of kynurenic acid and xanthurenic acid, and the intermediate preceding quinolinic acid, 3‐hydroxyanthranilic acid, did not differ from controls. It would appear that these pathways are prioritized over the main quinolinic acid synthesis pathway. It is not impossible to exclude a shift from the main pathway to picolinic acid as we were unable to measure this metabolite because the values obtained were below the detection limit for all samples. Interestingly, apart from Trp, our results are in line with a study investigating kynurenine metabolism in adult PKU, albeit including various forms of PKU [24]. In their study, they assumed accelerated catabolism of kynurenines, ruling out hypometabolism because they were unable to measure serotonin. Our results seem to agree with their hypothesis in view of the hypometabolism of serotonin. The hypothesis that there was an increase in quinolinic acid synthesis (not measured in their study) seems, however, to be ruled out by our results. This suggests that the increased catabolism of kynurenines is due to enhanced synthesis of kynurenic acid and xanthurenic acid, and potentially picolinic acid. A summary of these findings is shown in Figure 4.

In addition to these two pathways, Trp can also be metabolized to indole and its derivatives by the gut microbiota [63, 64]. Trp metabolism by the microbiota plays an important role in the synthesis of many aryl hydrocarbon receptor (AHR) ligands. Once bound to its ligand, AHR induces changes in the intestinal epithelium and inhibits inflammation [65]. Among those we were able to measure were 3‐indole acetic acid, 3‐indole‐aldehyde acid, and I3LA, all of which are agonists of AHR [66, 67]. Only I3LA is significantly different between patients and controls, on average about sevenfold higher in PKU patients. High I3LA concentrations in PKU have also been reported by Weerd et al. [30]. This Trp derivative can be synthesized by *lactobacillus* and *bifidobacterium* [68]. Interestingly, Mancilla et al. [69] observed an enrichment in *bifidobacterium* and a reduction in the presence of *lactobacillus*. These results appear to be consistent with our own, indicating a potential development of this *genus* in adult PKU. Although I3LA has many beneficial aspects via its effect on AHR, we are unable to ascertain whether this is the case at these supraphysiological concentrations. An in‐depth study of the microbiota of adult PKU with large sample sizes, coupled with a metabolomic study, could shed further light on these questions. The last Trp metabolite indirectly linked to the microbiota that could be measured in this study was indoxyl sulfate (IS). The microbiota metabolizes Trp to indole, which is then metabolized to IS in the liver [70] and to a lesser extent in colonocytes [71]. The latter is significantly reduced compared with controls, probably due to the low bioavailability of indole caused by excessive metabolization of Trp by the microbiota into I3LA.

### 5.3 Other Findings

#### 5.3.1 Reduced Glu Level

Glu was implicated in the most metabolic perturbations (Ala, Asp, Glu metabolism, Arg and Pro metabolism, Arg biosynthesis, glyoxylate and dicarboxylate metabolism, and butanoate metabolism). Moritz et al. [27] and Cannet et al. [25] reported an increase in Glu in metabolomic studies probably due to cohort differences in terms of dietary monitoring and age. Interestingly, one study suggested that Glu was of high diagnostic value in monitoring patients [72]. Another study of children with different degrees of phenylalaninaemia reported a Glu deficiency in patients with poor metabolic control compared to control that was not present in children with good metabolic control [73]. These results may explain our findings concerning this AA, given that the patients studied had poor metabolic control (1401 ± 434 μmol/L). On the other hand, several studies have shown that there is an increase in glutamyl‐Phe or glutamyl‐glutamyl‐Phe in adult PKU patients [11, 19, 31]. The formation of these oligopeptides could also play a role in the low Glu levels found in the patients studied. Another hypothesis could be a defect in the glutathione pathway due to severe oxidative stress. Indeed, glutamate is essential for glutathione synthesis (van der Pol et al. 2017). 5‐oxoproline, one of the degradation products of glutathione, is one of the only metabolites elevated compared with the control (fold change = 2.14) (van der Pol et al. 2017). Impaired glutathione metabolism is also well documented in PKU [53]. Given the central role of Glu in metabolism [74] and its possible responsiveness to diet, it would be interesting to monitor it in adult PKU.

#### 5.3.2 Energy Metabolism

In the present study, we found some intermediates of the TCA cycle, such as malic acid (fold change = 0.55), isocitric acid (fold change = 0.6) and one of the products of this cycle, lactate (fold change = 0.62). All these metabolites were less present in adult PKU, suggesting energy hypometabolism and mitochondrial dysfunction, which is a recognized feature of PKU [21, 22]. Moritz et al. [27] reported a citric acid deficiency in plasma, potentially linked to a disturbance in energy metabolism. On the other hand, Cannet et al. [25] reported an increase in this metabolite during the study of a cohort of adults in the same matrix. These findings on energy metabolism were also found to be associated with oxaloacetic acid deficiency in the urine of patients [26] and via succinic acid deficiency in the same matrix [29]. Citrate (fold change = 0.6) and succinic acid (fold change = 0.52) are also at reduced levels in our study. It is also important to note that reduced Glu levels could be linked to alterations in the TCA cycle. Glu can be converted to alpha‐ketoglutarate by an anaplerotic reaction to compensate for the lack of one of the intermediates of the cycle; the latter also being less present in adult PKU (fold change = 0.67). The butanoate metabolism identified by the OPLSDA model is also linked to energy metabolism due to the low level of acetoacetic acid (fold change = 0.55) which is a precursor of acetyl‐CoA, and the low level of Glu, which could be used to generate a TCA cycle intermediate as indicated above. Glyoxylate and dicarboxylate metabolism has also been identified in the OPLSDA model due to low levels of several metabolites of the TCA cycle, Glu, as well as low levels of glyoxylate (fold change = 0.66). In humans, glyoxylate may be derived from mitochondrial degradation of 4‐hydroxyproline (degradation product of collagen, elastin…), leading to the synthesis of glyoxylate and pyruvate [75]. Glyoxylate can also be degraded to Gly and pyruvate in peroxisomes [75]. Could excessive glyoxylate use relative to control be a compensatory mechanism to generate material for the TCA cycle to maintain efficient energy metabolism? Further large‐scale studies in PKU, focusing on these metabolisms, are needed to test these hypotheses.

Our study has some strengths and limitations. The main strength of our study is the exclusive inclusion of adult patients with classic PKU, the use of LC/MS complemented by a multimodal approach (polar, apolar, and lipids) which allows the detection of an increased number of metabolites supplemented by a quantitative approach for Trp metabolites, including some compounds not yet measured in PKU. The limitations of the present study are the small sample size of patients and controls that we included, as well as the inclusion of both patients with or without PKU treatment (some patients had some AAs supplements, and some have not). We did not consider this parameter because of the poor metabolic control within the cohort and the loss of statistical power by subdividing the patients' group. Furthermore, none of the metabolites differed significantly between patients on and off diet. It would nevertheless be of interest to consider it in future research involving a large number of patients with varying degrees of metabolic control. The question of the influence of AA supplements on plasma Tyr concentrations can also be questioned, although such influence may be small, as Tyr was measured at the fasting state [3].

In summary, the multimodal metabolomic analysis of the present study has brought to light as yet undescribed metabolic pathways in adult PKU, as well as a set of metabolites common to the different models which together constitute excellent leads for further research into the pathophysiology of adult PKU. The analysis focused on Trp has enabled us to deepen our understanding of the disruption of its metabolism in adult PKU, which may have an impact on inflammation, energy metabolism, and neurotransmission, and to which the microbiota may contribute. Further studies are needed to determine the contribution of the identified pathways in the pathophysiology of adult PKU, as well as for the disruption of Trp metabolism and the involvement of the microbiota in it.

## Ethics Statement

The present study has been conducted in accordance with the ethical standards on human experimentation of our institution and with the Helsinki declaration of 1975, revised in 2013. (comment: the French committee of persons protection is the ethic committee).

## Conflicts of Interest

F. Maillot has been a member of advisory boards for PTC Therapeutics and Jnana Therapeutics, has received payment for a lecture from PTC Therapeutics, has received a research grant from BioMarin Pharmaceutical, and has received support for attending meetings and/or travel from Sanofi and Takeda. The other authors declare no conflicts of interest.

## Supporting information


**Data S1.** Supporting Information.

## Data Availability

The data that support the findings of this study are available from the corresponding author upon reasonable request.
