# Supplementary material for: Multimodal Metabolomic Analysis Reveals Novel Metabolic Disturbances in Adults With Early Treated Phenylketonuria
Source: JIMD Rep. 2025 Mar 24;66(2):e70010. doi: 10.1002/jmd2.70010 (PMC11932803; doi:10.1002/jmd2.70010)
Supplement: Supplementary file 1 — Data S1. Supporting Information. [file JMD2-66-e70010-s001.zip › Figure_S2_common_metabolites.pptx]

## Slide 1
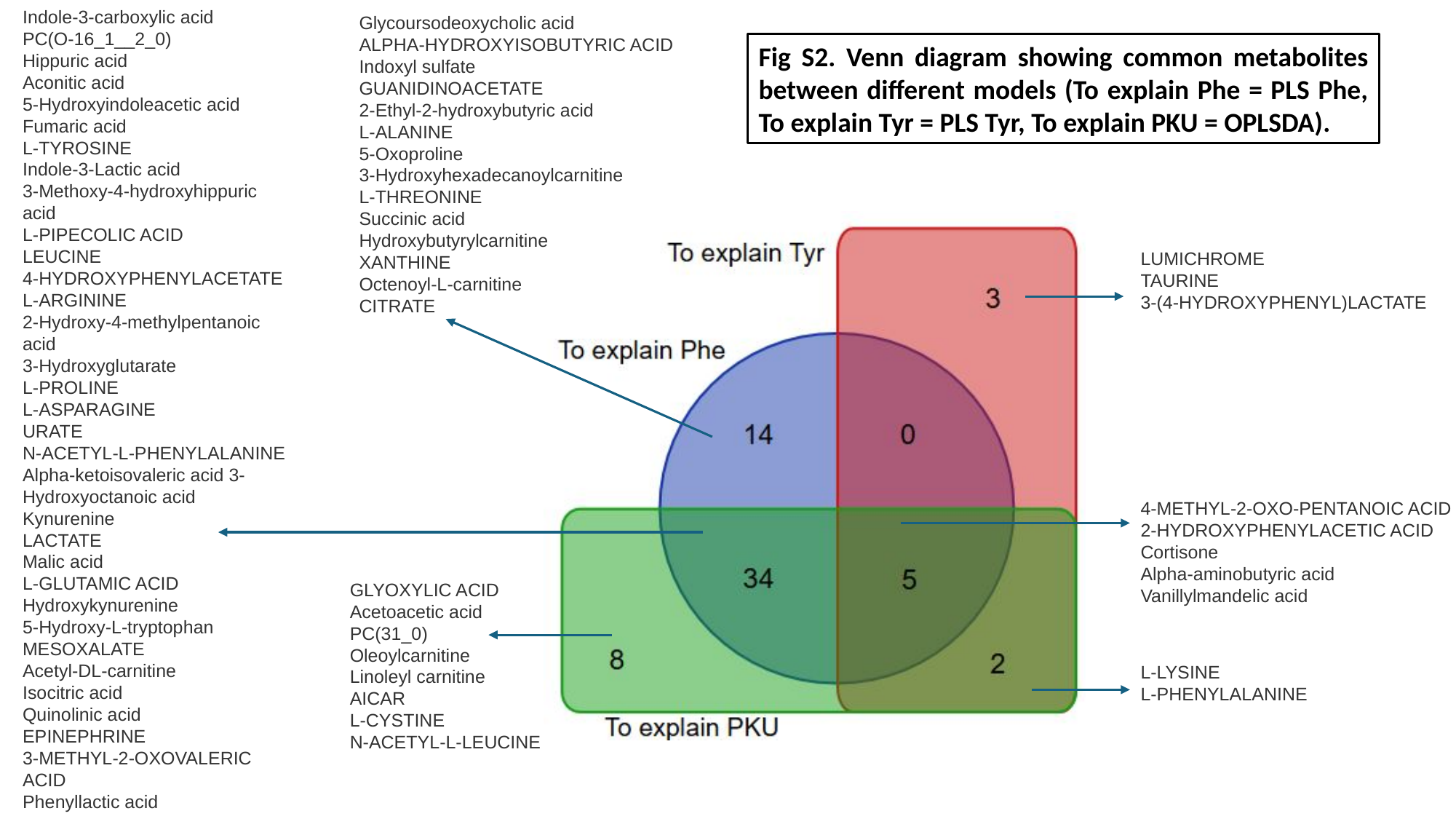

Indole-3-carboxylic acid
PC(O-16_1__2_0)
Hippuric acid
Aconitic acid
5-Hydroxyindoleacetic acid
Fumaric acid
L-TYROSINE
Indole-3-Lactic acid
3-Methoxy-4-hydroxyhippuric acid
L-PIPECOLIC ACID
LEUCINE
4-HYDROXYPHENYLACETATE
L-ARGININE
2-Hydroxy-4-methylpentanoic acid
3-Hydroxyglutarate
L-PROLINE
L-ASPARAGINE
URATE
N-ACETYL-L-PHENYLALANINE
Alpha-ketoisovaleric acid 3-
Hydroxyoctanoic acid
Kynurenine
LACTATE
Malic acid
L-GLUTAMIC ACID
Hydroxykynurenine
5-Hydroxy-L-tryptophan
MESOXALATE
Acetyl-DL-carnitine
Isocitric acid
Quinolinic acid
EPINEPHRINE
3-METHYL-2-OXOVALERIC ACID
Phenyllactic acid
Glycoursodeoxycholic acid
ALPHA-HYDROXYISOBUTYRIC ACID
Indoxyl sulfate
GUANIDINOACETATE
2-Ethyl-2-hydroxybutyric acid
L-ALANINE
5-Oxoproline
3-Hydroxyhexadecanoylcarnitine
L-THREONINE
Succinic acid
Hydroxybutyrylcarnitine
XANTHINE
Octenoyl-L-carnitine
CITRATE
Fig S2. Venn diagram showing common metabolites between different models (To explain Phe = PLS Phe, To explain Tyr = PLS Tyr, To explain PKU = OPLSDA).
LUMICHROME
TAURINE
3-(4-HYDROXYPHENYL)LACTATE
4-METHYL-2-OXO-PENTANOIC ACID
2-HYDROXYPHENYLACETIC ACID
Cortisone
Alpha-aminobutyric acid
Vanillylmandelic acid
GLYOXYLIC ACID
Acetoacetic acid
PC(31_0)
Oleoylcarnitine
Linoleyl carnitine
AICAR
L-CYSTINE
N-ACETYL-L-LEUCINE
L-LYSINE
L-PHENYLALANINE
