# Supplementary material for: Multimodal Metabolomic Analysis Reveals Novel Metabolic Disturbances in Adults With Early Treated Phenylketonuria
Source: JIMD Rep. 2025 Mar 24;66(2):e70010. doi: 10.1002/jmd2.70010 (PMC11932803; doi:10.1002/jmd2.70010)
Supplement: Supplementary file 1 — Data S1. Supporting Information. [file JMD2-66-e70010-s001.zip › Figure_S3_Model_with_dietary_data.pptx]

## Slide 1
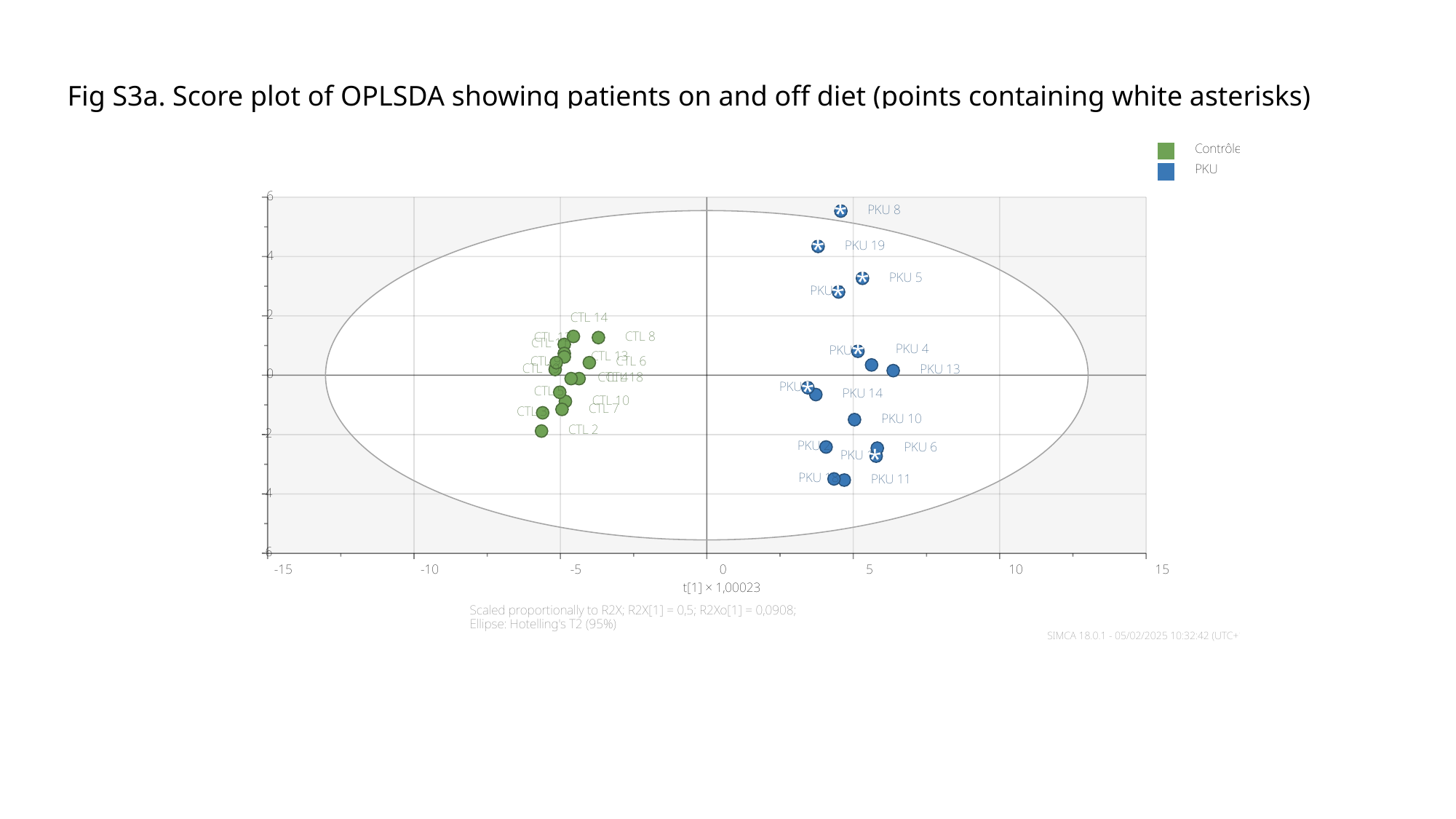

Fig S3a. Score plot of OPLSDA showing patients on and off diet (points containing white asterisks)
*
*
*
*
*
*
*

## Slide 2
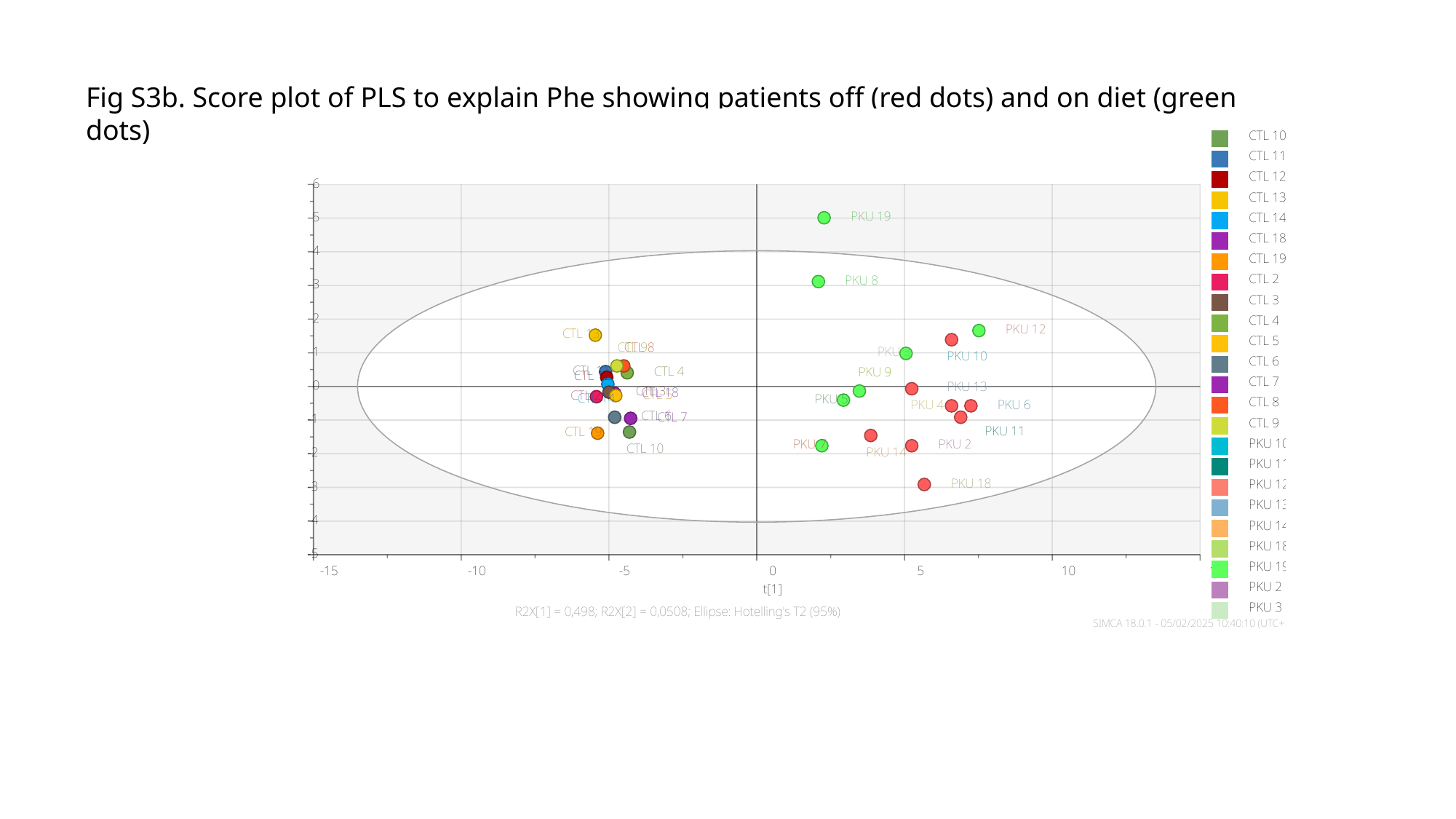

Fig S3b. Score plot of PLS to explain Phe showing patients off (red dots) and on diet (green dots)

## Slide 3
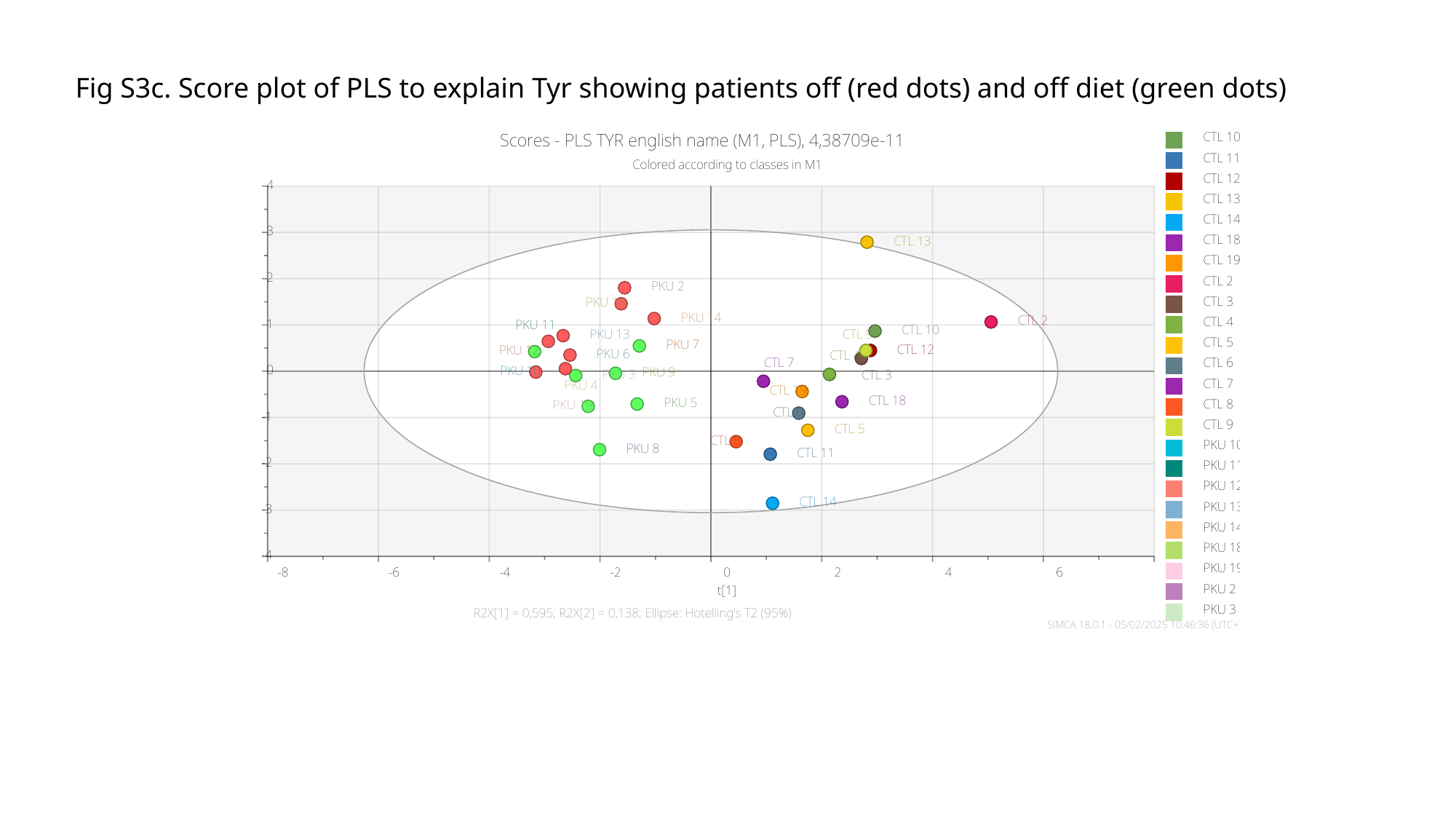

Fig S3c. Score plot of PLS to explain Tyr showing patients off (red dots) and off diet (green dots)
