# Supplementary figures and images for: Multimodal Metabolomic Analysis Reveals Novel Metabolic Disturbances in Adults With Early Treated Phenylketonuria
Source: JIMD Rep. 2025 Mar 24;66(2):e70010. doi: 10.1002/jmd2.70010 (PMC11932803; doi:10.1002/jmd2.70010)

## Slide 1
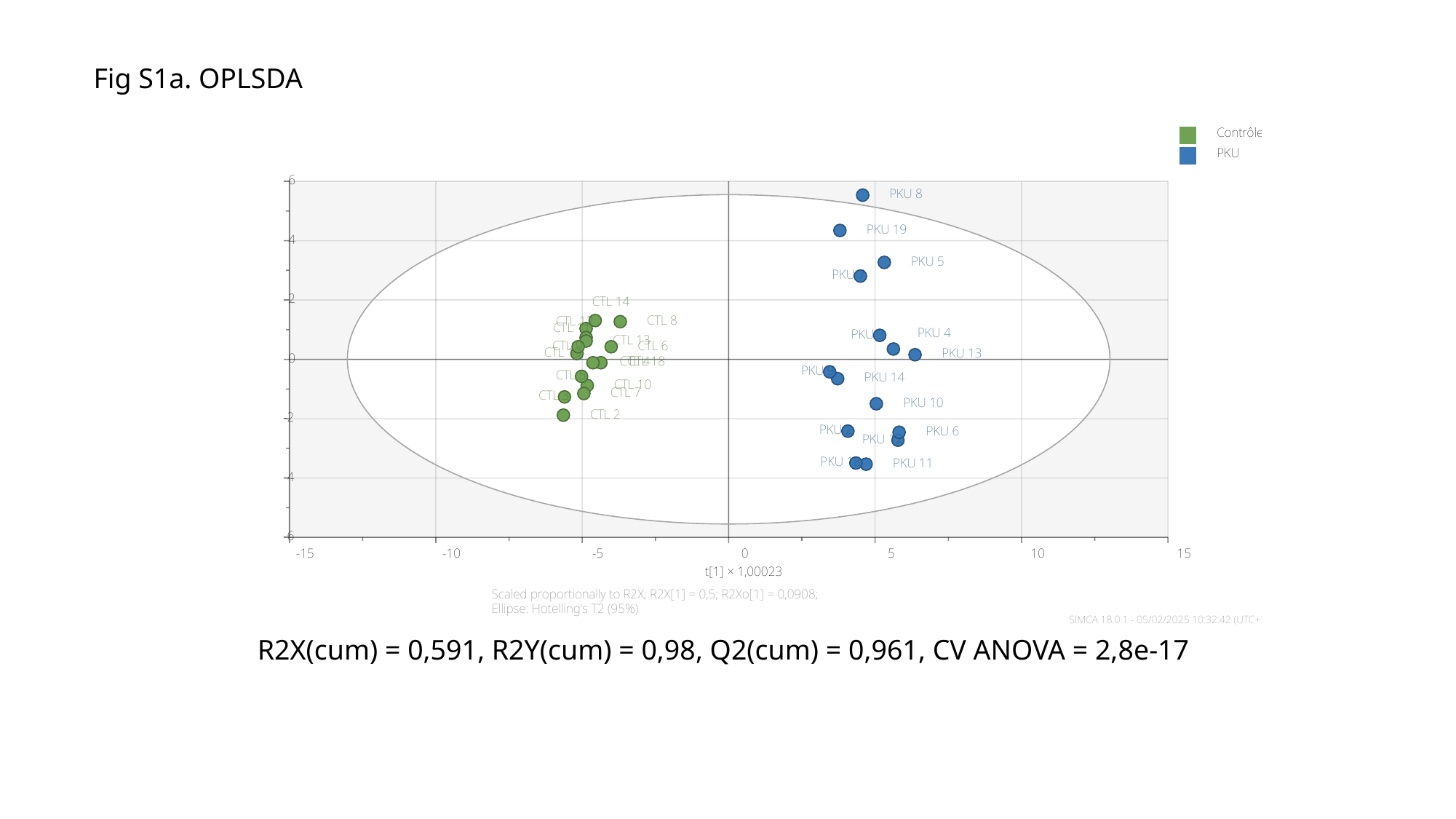

Fig S1a. OPLSDA
R2X(cum) = 0,591, R2Y(cum) = 0,98, Q2(cum) = 0,961, CV ANOVA = 2,8e-17

## Slide 2
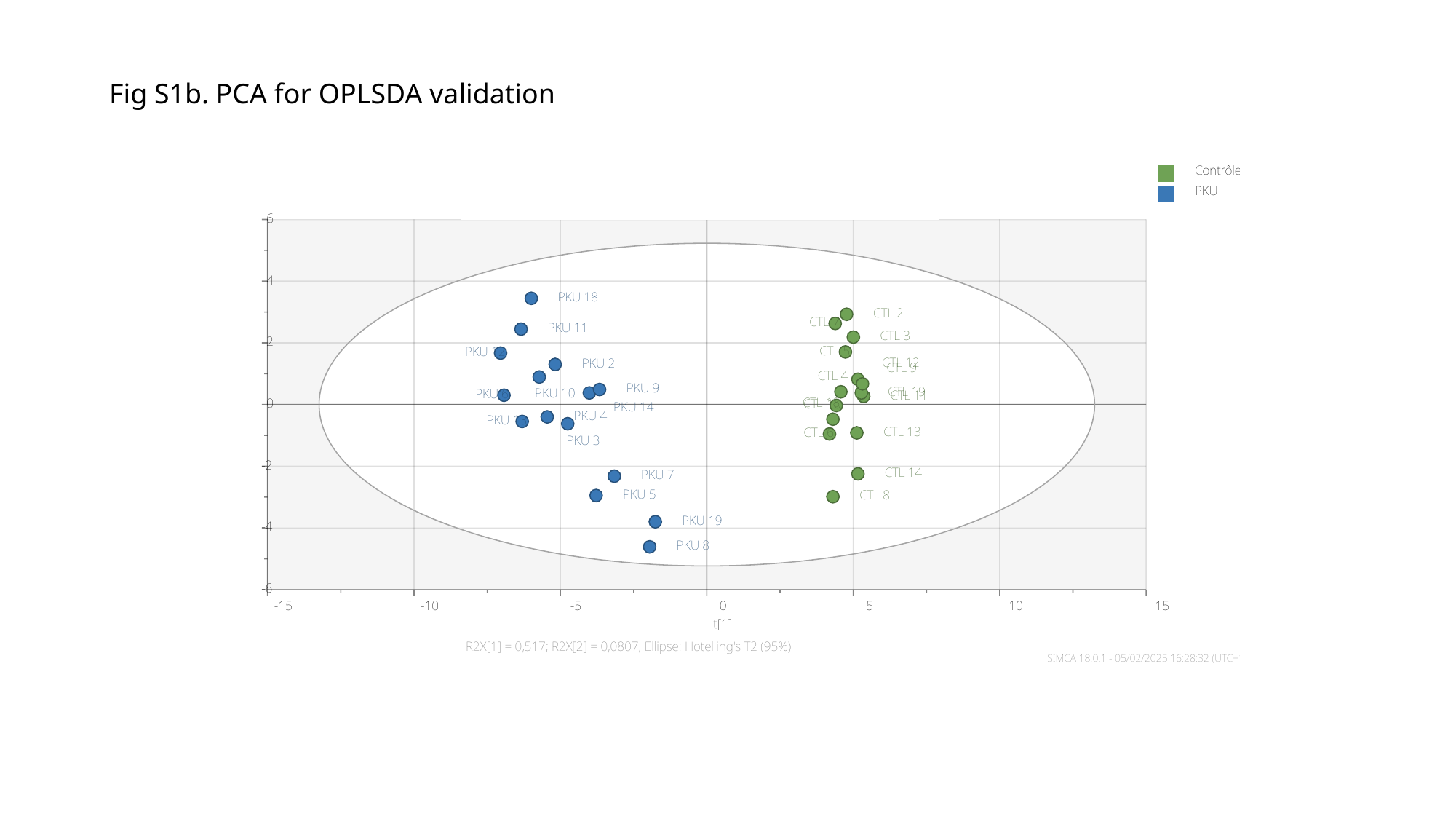

Fig S1b. PCA for OPLSDA validation

Supplement: Supplementary file 1 — Data S1. Supporting Information. [file JMD2-66-e70010-s001.zip › Figure_S1_OPLSDA.pptx]
